# Integration of HIV care into maternal and child health services in the global IeDEA consortium

**DOI:** 10.3389/fgwh.2023.1066297

**Published:** 2023-04-17

**Authors:** John Humphrey, Elizabeth Nagel, James G. Carlucci, Andrew Edmonds, Aarti Kinikar, Kim Anderson, Valériane Leroy, Daisy Machado, Dwight E. Yin, Marco Tulio Luque, Madeleine Amorissani-Folquet, Safari Mbewe, Tulathip Suwanlerk, Athanase Munyaneza, Rena C. Patel, Beverly Musick, Lisa Abuogi, Kara Wools-Kaloustian

**Affiliations:** ^1^ Department of Medicine, Indiana University, Indianapolis, Indiana, IN, United States; ^2^ Department of Epidemiology, The University of North Carolina at Chapel Hill, Chapel Hill, NC, United States; ^3^ Department of Pediatrics, Byramjee Jeejeebhoy Government Medical College, Pune, India; ^4^ Centre for Infectious Disease Epidemiology and Research, Faculty of Health Sciences, School of Public Health and Family Medicine, University of Cape Town, Cape Town, South Africa; ^5^ CERPOP- UMR 1295, Institut National de la Santé et de la Recherche Médicale, University Toulouse 3, France; ^6^ Departamento de Pediatria, Escola Paulista de Medicina, Universidade Federal de São Paulo, São Paulo, Brazil; ^7^ Maternal Adolescent and Pediatric Research Branch (MAPRB), Division of AIDS (DAIDS), Prevention Sciences Program (PSP), National Institute of Allergy and Infectious Diseases, National Institutes of Health, Rockville, MD, United States; ^8^ Departamento de Pediatría, Instituto Hondureño de Seguridad Social and Hospital Escuela Universitario, Tegucigalpa, Honduras; ^9^ CHU Cocody, University Houphouet Boigny, Abidjan, Côte d’Ivoire; ^10^ Lighthouse Trust, Lilongwe, Malawi; ^11^ TREAT Asia/amfAR—The Foundation for AIDS Research, Bangkok, Thailand; ^12^ Research for Development (RD Rwanda) and Rwanda Military Hospital, Kigali, Rwanda; ^13^ Department of Medicine, University of Washington, Seattle, WA, United States; ^14^ Department of Pediatrics, University of Colorado, Aurora, CO, United States

**Keywords:** decentralization, pregnant women, postpartum, infant, service & deployment models, HIV service delivery, care model

## Abstract

The WHO recommends the integration of routine HIV services within maternal and child health (MCH) services to reduce the fragmentation of and to promote retention in care for pregnant and postpartum women living with HIV (WWH) and their infants and children exposed to HIV (ICEH). During 2020–2021, we surveyed 202 HIV treatment sites across 40 low- and middle-income countries within the global International epidemiology Databases to Evaluate AIDS (IeDEA) consortium. We determined the proportion of sites providing HIV services integrated within MCH clinics, defined as full [HIV care *and* antiretroviral treatment (ART) initiation in MCH clinic], partial (HIV care *or* ART initiation in MCH clinic), or no integration. Among sites serving pregnant WWH, 54% were fully and 21% partially integrated, with the highest proportions of fully integrated sites in Southern Africa (80%) and East Africa (76%) compared to 14%–40% in other regions (i.e., Asia-Pacific; the Caribbean, Central and South America Network for HIV Epidemiology; Central Africa; West Africa). Among sites serving postpartum WWH, 51% were fully and 10% partially integrated, with a similar regional integration pattern to sites serving pregnant WWH. Among sites serving ICEH, 56% were fully and 9% were partially integrated, with the highest proportions of fully integrated sites in East Africa (76%), West Africa (58%) and Southern Africa (54%) compared to ≤33% in the other regions. Integration was heterogenous across IeDEA regions and most prevalent in East and Southern Africa. More research is needed to understand this heterogeneity and the impacts of integration on MCH outcomes globally.

## Introduction

The World Health Organization (WHO) defines HIV integration as “the co-location and sharing of services and resources across different health areas and includes offering HIV testing, prevention, treatment and care services alongside other relevant health services” (1). In 2006, the WHO recommended the integration of routine HIV services within maternal and child health (MCH) services as a strategy to reduce the fragmentation of and promote retention in care for pregnant and postpartum women living with HIV (WWH) and their infants (2, 3). Limited evidence from low- and middle-income countries (LMICs) suggests that integration improves outcomes for these populations, including uptake of maternal and infant HIV testing and antiretroviral treatment (ART), retention in care, and care quality (4–10). Integration may also facilitate longitudinal HIV testing and early diagnosis for infants and children exposed to HIV (ICEH), which is particularly critical in settings such as sub-Saharan Africa where routine breastfeeding may last 24 months or more (11). Adverse effects of integration have also been reported, and include heavier provider workloads, increased patient wait times, insufficient clinic space and resources, the risk of unwanted HIV status disclosure, and the need to revise monitoring and evaluation tools and reporting systems for pregnant WWH and their infants and children exposed to HIV (ICEH) (12–16). Moreover, while some have advocated for integration as a strategy to enhance program efficiency, particularly in the context of declining donor funding for HIV, data supporting the cost-effectiveness of integration have been mixed, in part because per-patient cost savings associated with integration are offset by the resources needed for implementation (15). Consequently, nearly two decades after the WHO first recommended the integration of HIV and MCH services, the WHO evidence grade for this recommendation remains very-low-certainty (1).

Despite this uncertainty, there is a growing body of literature indicating that integrated HIV and MCH services are increasingly being adopted in LMICs, particularly in sub-Saharan Africa where the burden of HIV is highest (15). Yet while these studies suggest that HIV services are more commonly integrated with MCH services compared to other health services, the extent to which HIV care and treatment programs in LMICs have adopted HIV and MCH integration globally is unknown (4, 9, 15). The ways that HIV and MCH integration is configured within programs is also largely unknown and likely heterogeneous, and may include HIV testing, ART initiation for patients newly diagnosed with HIV, or continuation of ART for established patients. Understanding the current epidemiology and implementation of integrated HIV and MCH services is needed to guide research on the drivers and outcomes of HIV and MCH integration in LMICs.

The International epidemiology Databases to Evaluate AIDS (IeDEA) is a research consortium established by the U.S. National Institutes of Health which pools and harmonizes HIV data from over two million people with and at risk of HIV at clinical sites in 44 countries around the world (www.iedea.org). IeDEA has conducted periodic consortium-wide site assessment surveys describing the available resources and implementation of HIV care and treatment for people living with HIV at affiliated sites since 2009 (17, 18). Data from these surveys are used to document site-level capacity and availability of services critical to understanding global trends in HIV care and treatment implementation, as such data are not usually available in routine program databases. IeDEA's latest site assessment was implemented in 2020–2021, offering a unique opportunity to assess the uptake of integrated HIV and MCH services within LMICs globally. The objective of this study was to determine the prevalence and characteristics of integrated HIV and MCH services at LMIC sites participating in the global IeDEA consortium.

## Methods

### Setting and study population

The IeDEA site assessment was a cross-sectional survey of all clinical sites that contributed longitudinal patient data to IeDEA in 2020. These sites represent seven geographic regions: Asia-Pacific, the Caribbean, Central and South America Network for HIV Epidemiology (CCASAnet), North America (i.e., The North American AIDS Cohort Collaboration on Research and Design, NA-ACCORD), and four sub-Saharan Africa regions (Central, East, Southern, and West Africa). As the Southern Africa region included >200 small clinics contributing data to IeDEA as part of larger programmatic cohorts, it was not considered feasible to survey 100% of active sites in this region. Therefore, a systematic, hybrid sampling strategy was used to (i) purposefully sample sites that had completed prior IeDEA site surveys to facilitate future longitudinal analyses, and (ii) ensure that selected sites were representative of the larger programmatic cohort by randomly sampling sites stratified by location (urban vs. rural) in line with the clinic distribution within each programmatic cohort. Thus, 32 representative sites from the Southern Africa region (15% of active sites) were included in the site assessment, which included sites that were purposively sampled (*n* = 5), randomly sampled (*n* = 18), and those in IeDEA's database that are not part of a large programmatic cohort (*n* = 9) (19).

Sites in IeDEA regions were eligible for this analysis if they reported providing HIV services to pregnant WWH, postpartum WWH, or ICEH <24 months of age. NA-ACCORD was not included in the analysis as it comprises high-income countries with low HIV burdens, where factors influencing the integration of HIV and MCH services may differ markedly from those in LMICs. Sites and coordinating centers for all regions had institutional review board approvals in place permitting the collection of site-level data for this survey. Specifically, this study was approved by the Vanderbilt University Institutional Review Board where the survey data are housed.

### Data collection and management

Survey development and data collection methods have been previously described (18). Briefly, IeDEA investigators developed the survey based on prior IeDEA-wide surveys and input from technical working groups. Core domains of the survey that pertained to this study included patient populations served, HIV testing, and HIV care and treatment. Questions about the integration of HIV and MCH services were also incorporated into the survey and included the locations of HIV care and ART initiation for pregnant WWH, postpartum WWH, and ICEH. These location response options were not mutually exclusive, such that respondents were allowed to indicate more than one location of HIV care and ART initiation for each population at their clinic.

Though originally intended to assess service delivery at the time the survey was administered in 2020–2021, the year 2019 was selected as the reference period for all survey questions due to the potential for disruptions in service delivery related to the COVID-19 pandemic. The survey was programmed in REDCap and distributed using a web-based link to a designated clinical staff member for each IeDEA clinic or cohort of clinics. Some sites completed paper-based surveys, which were entered into REDCap. The accuracy of data input was verified by the regional data team. REDCap servers were housed in a local data center hosted at Vanderbilt University Medical Center. Data from English and French surveys were merged in REDCap and exported for analysis.

To inform our interpretation of the data, we also conducted a non-systematic search of the literature (PubMed, Google scholar) and ministry of health websites for policies recommending the integration of HIV and MCH services on or before 2019 in each country included in the IeDEA survey. For this search, the presence of integration policy was defined as any statement recommending the delivery of HIV care or ART initiation in MCH clinic from a ministry of health or other government entity. The search was facilitated and verified by the study coauthors representing each IeDEA region. Countries for which integration policy was not identified in our search were designated as having “unconfirmed” integration policy to acknowledge the potential limitations of our non-systematic search. The results of the search were descriptively summarized and presented in the Supplementary Material (Supplementary Table S1).

### Statistical analysis

Site characteristics relevant to service delivery for MCH clients were descriptively summarized overall, by IeDEA region, and by population setting (urban, rural, or mixed urban/rural). The primary outcome was the proportion of sites providing HIV services integrated within MCH clinics for pregnant WWH, postpartum WWH, and ICEH, respectively. For pregnant WWH, this included integration of HIV services within antenatal care (ANC) clinics; for postpartum WWH, this included integration of HIV services within postnatal care (PNC) clinics (i.e., clinics for postpartum women only) or MCH clinics (i.e., clinics for women and infants); and, for ICEH, this included integration of HIV services within well-baby clinics (i.e., clinics for infants) or MCH clinics. Integration was defined as full (integration of HIV care *and* ART initiation), partial (integration of HIV care *or* ART initiation), and no integration. These definitions were based on WHO guidance (1, 20). Tableau (Tableau Software, LLC, Seattle, WA) was used to map the IeDEA sites included in the analysis, and SAS version 9.4 (SAS Institute Inc., Cary, NC) was used for analysis.

## Results

**Figure 1:**
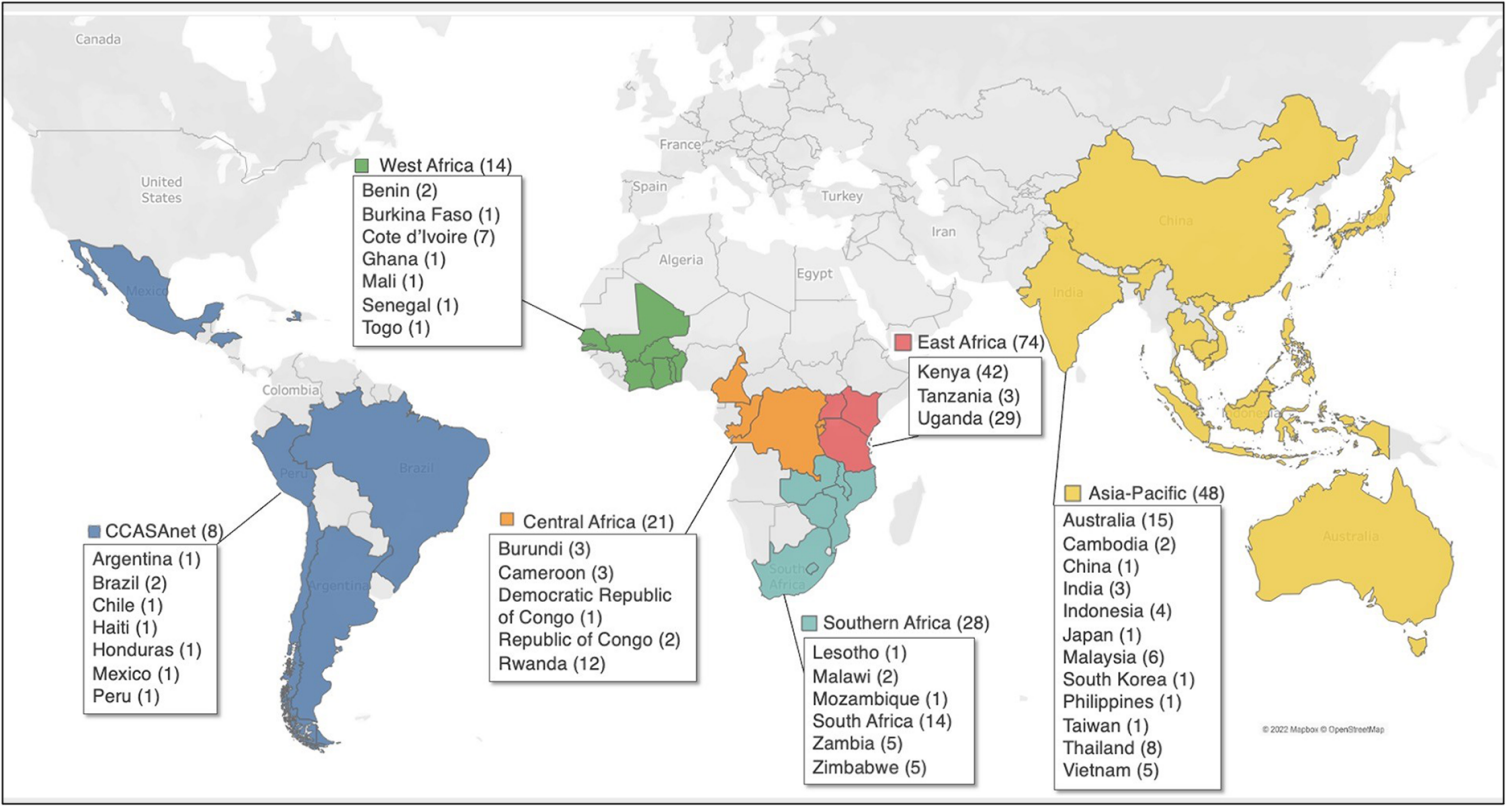
IeDEA sites included in the analysis.

Between September 2020 and March 2021, clinical staff at 202 sites in 40 LMICs completed the survey, and of these sites, 193 (96%) reported providing services to pregnant WWH, postpartum WWH, or ICEH and were included in the analysis (Figure 1). The number of sites included in the analysis in each IeDEA region were: Asia-Pacific (*n* = 48), CCASAnet (*n* = 8), Central Africa (*n* = 21), East Africa (*n* = 74), Southern Africa (*n* = 28) and West Africa (*n* = 14).

Overall, 58 (30%) sites served an urban population, 43 (22%) served a rural population, and the remainder served a mixed urban/rural population (Table 1). Most sites reported providing services to pregnant WWH (92%), postpartum WWH (88%), or ICEH (82%), while fewer sites (73%) provided services to all three groups. Pregnancy/breastfeeding services and ICEH services were available every day, as opposed to special/dedicated days, in 60% and 74% of sites, respectively. HIV DNA or RNA PCR for infant HIV diagnosis testing was available at 81% of sites, ranging from >85% in East, Southern and West Africa to 54%–75% in the other regions.

**Table 1:** Characteristics of service delivery for pregnant and postpartum women with HIV and infants with perinatal HIV exposure, by IeDEA region, September 2020 to March 2021.

Characteristic	Asia-Pacific *N* = 48 *N* (%)	CCASAnet *N* = 8 *N* (%)	Central Africa *N* = 21 *N* (%)	East Africa *N* = 74 *N* (%)	Southern Africa *N* = 28 *N* (%)	West Africa *N* = 14 *N* (%)	Total *N* = 193 *N* (%)
Setting							
Urban	19 (40)	8 (100)	7 (33)	1 (1)	16 (57)	7 (50)	58 (30)
Rural	1 (2)	0 (0)	2 (10)	37 (50)	3 (11)	0 (0)	43 (22)
Mixed urban/rural	28 (58)	0 (0)	12 (57)	36 (49)	9 (32)	7 (50)	92 (48)
Population served^a^							
Pregnant WWH	42 (88)	7 (88)	20 (95)	74 (100)	25 (89)	10 (71)	178 (92)
Postpartum WWH	37 (77)	8 (100)	17 (81)	72 (97)	25 (89)	10 (71)	169 (88)
ICEH	25 (52)	6 (75)	20 (95)	72 (97)	24 (86)	12 (86)	159 (82)
Pregnant/postpartum WWH	35 (73)	7 (88)	17 (81)	72 (97)	24 (86)	9 (64)	164 (85)
Pregnant/postpartum WWH and ICEH	18 (38)	6 (75)	17 (81)	70 (95)	21 (75)	8 (57)	140 (73)
Pregnancy/breastfeeding services available^a^							
Every clinic day	23 (48)	6 (75)	15 (71)	47 (64)	17 (61)	8 (57)	116 (60)
Special/dedicated days	9 (19)	1 (13)	7 (33)	25 (34)	5 (18)	0 (0)	47 (24)
Not available	16 (33)	1 (13)	0 (0)	3 (4)	6 (21)	6 (43)	32 (17)
ICEH services available							
Every clinic day	18 (37)	5 (62)	15 (71)	70 (95)	23 (85)	12 (92)	143 (74)
Special/dedicated days	8 (17)	1 (13)	5 (24)	4 (5)	1 (4)	0 (0)	19 (10)
Not available	22 (46)	2 (25)	1 (5)	0 (0)	3 (11)	1 (8)	29 (15)
HIV DNA or RNA PCR for infant HIV diagnosis	26 (54)	6 (75)	19 (91)	71 (96)	24 (86)	10 (71)	156 (81)

ICEH, infants and children exposed to HIV; PCR, polymerase chain reaction; WWH, women living with HIV.

^a^ Totals exceed 100% in some region because responses were not mutually exclusive.

For pregnant WWH, HIV care was most frequently delivered in the ANC clinic in 80% of sites in East and Southern Africa (Table 2). However, 84% of sites in Southern Africa also reported delivering HIV care to pregnant WWH in the HIV clinic, compared to 19% of sites in East Africa. As with Southern Africa, other regions had similar proportions of sites providing HIV care to pregnant WWH in HIV and ANC clinics. Among all sites, ART initiation for pregnant WWH was less frequently offered in ANC clinics (59%) compared to HIV clinics (71%). Full and partial integration of services for pregnant WWH occurred in 54% and 21% of sites, respectively, with the highest proportions of fully integrated sites reported in Southern Africa (80%) and East Africa (76%).

**Table 2:** Integration of HIV and maternal and child health services, by IeDEA region, September 2020 to March 2021.

Characteristic	Asia-Pacific *N* (%)	CCASAnet *N* (%)	Central Africa *N* (%)	East Africa *N* (%)	Southern Africa *N* (%)	West Africa *N* (%)	Total *N* (%)
Pregnant WWH	*N* = 42	*N* = 7	*N* = 20	*N* = 74	*N* = 25	*N* = 10	*N* = 178
HIV care							
HIV clinic	38 (90)	4 (57)	13 (65)	14 (19)	21 (84)	7 (70)	97 (55)
ANC clinic	25 (60)	5 (71)	10 (50)	59 (80)	20 (80)	7 (70)	126 (71)
Other	2 (5)	0 (0)	2 (10)	8 (11)	1 (4)	0 (0)	13 (7)
ART initiation							
HIV clinic	38 (90)	6 (86)	15 (75)	16 (22)	10 (40)	7 (70)	92 (52)
ANC clinic	11 (26)	1 (14)	9 (45)	58 (78)	21 (84)	5 (50)	105 (59)
Other	3 (7)	0 (0)	2 (10)	7 (9)	1 (4)	0 (0)	13 (7)
Integration status							
Full	10 (24)	1 (14)	6 (30)	56 (76)	20 (80)	4 (40)	97 (54)
Partial	16 (38)	4 (57)	7 (35)	5 (7)	1 (4)	4 (40)	37 (21)
None	16 (38)	2 (29)	7 (35)	13 (17)	4 (16)	2 (20)	44 (25)
Postpartum WWH	*N* = 37	*N* = 8	*N* = 17	*N* = 72	*N* = 25	*N* = 10	*N* = 169
HIV care							
HIV clinic	33 (89)	7 (88)	13 (76)	18 (25)	12 (48)	8 (80)	91 (54)
PNC	7 (19)	2 (25)	2 (12)	11 (15)	11 (44)	1 (10)	34 (20)
MCH clinic	7 (19)	0 (0)	8 (47)	55 (76)	13 (52)	3 (30)	86 (51)
Other	4 (11)	0 (0)	1 (6)	8 (11)	0 (0)	0 (0)	13 (8)
ART initiation							
HIV clinic	33 (89)	8 (100)	15 (88)	14 (19)	13 (52)	6 (60)	89 (53)
PNC	4 (11)	1 (13)	2 (12)	14 (19)	10 (40)	0 (0)	31 (18)
MCH clinic	3 (8)	1 (13)	8 (47)	55 (76)	11 (44)	1 (10)	79 (47)
Other	4 (11)	0 (0)	1 (6)	8 (11)	1 (4)	3 (30)	17 (10)
Integration status							
Full	5 (14)	1 (13)	8 (47)	55 (76)	16 (64)	1 (10)	86 (51)
Partial	6 (16)	2 (25)	2 (12)	2 (3)	1 (4)	3 (30)	16 (9)
None	26 (70)	5 (63)	7 (41)	15 (21)	8 (32)	6 (60)	67 (40)
ICEH	*N* = 25	*N* = 6	*N* = 20	*N* = 72	*N* = 24	*N* = 12	*N* = 159
HIV care							
HIV clinic	21 (84)	5 (83)	17 (85)	17 (24)	9 (38)	8 (67)	77 (49)
Well-baby clinic	10 (40)	1 (17)	1 (5)	11 (15)	7 (29)	7 (58)	37 (23)
MCH clinic	6 (24)	2 (33)	7 (35)	56 (78)	15 (63)	1 (8)	87 (55)
Other	4 (16)	0 (0)	1 (5)	8 (11)	2 (8)	0 (0)	15 (9)
ART initiation							
HIV clinic	20 (80)	5 (83)	19 (95)	24 (33)	13 (54)	9 (75)	90 (57)
Well-baby clinic	8 (32)	1 (17)	2 (10)	8 (11)	5 (21)	7 (58)	31 (20)
MCH clinic	2 (8)	2 (33)	3 (15)	54 (75)	13 (54)	3 (25)	77 (49)
Other	4 (16)	0 (0)	1 (5)	5 (7)	3 (13)	0 (0)	13 (8)
Integration status							
Full	8 (32)	2 (33)	4 (20)	55 (76)	13 (54)	7 (58)	89 (56)
Partial	4 (16)	0 (0)	3 (15)	4 (6)	5 (21)	0 (0)	16 (10)
None	13 (52)	4 (67)	13 (65)	13 (18)	6 (25)	5 (42)	54 (34)

ANC, antenatal care; ICEH, infants and children exposed to HIV; MCH, maternal and child health; PNC, postnatal clinic; WWH, women living with HIV.

For postpartum WWH, HIV care was delivered in the MCH clinic in 51% of sites overall and was highest in East Africa (76%). In contrast, approximately 80% of sites in the Asia-Pacific, CCASAnet, and Central and West Africa delivered HIV care in the HIV clinic. In Southern Africa, the location of HIV care for postpartum WWH was approximately 50% in each of the HIV, PNC, and MCH clinics. The locations of ART initiation for postpartum WWH (i.e., at the HIV, well-baby, MCH or other clinic) generally mirrored the locations of HIV care for postpartum WWH in each region. Full and partial integration of services for postpartum WWH occurred in 51% and 9% of sites, respectively, with the highest proportions of fully integrated sites in East Africa (76%) and Southern Africa (64%).

For ICEH, HIV care was delivered in the MCH clinic at 55% of sites overall and was highest in East Africa (78%) followed by Southern Africa (63%). Compared to the MCH clinic, the well-baby clinic accounted for a higher proportion of HIV care in the Asia-Pacific (40% vs. 24%) and West Africa (58% vs. 8%). The locations of ART initiation for ICEH diagnosed with HIV were generally the same as the locations of HIV care in each region. Full and partial integration of services for ICEH occurred in 56% and 10% of sites, respectively, with the highest proportions of fully integrated sites in East Africa (76%), West Africa (58%) and Southern Africa (54%).

**Figure 2:**
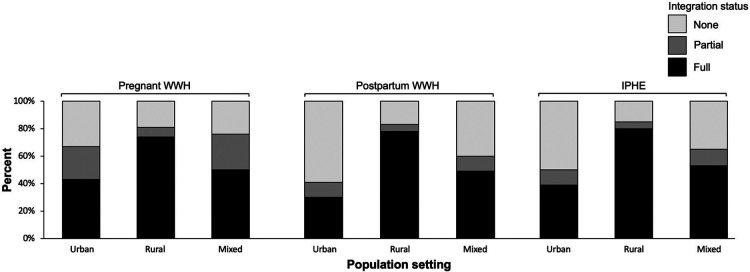
Integration of HIV and maternal and child health services, by urban and rural population setting, September 2020 to March 2021.

Stratifying the integration outcome by population setting, full integration for pregnant WWH (74%), postpartum WWH (78%), and ICEH (80%) was more prevalent in rural settings compared to 30%–53% of urban and mixed urban/rural settings (Figure 2; see Supplementary Table S1).

We found policies recommending integration for 31 of 40 (78%) countries, including at least one country in each IeDEA region (Supplementary Table S2). The proportions of countries with integration policy, by region, were: Asia Pacific (12/12, 100%), CCASAnet (2/7, 29%), Central Africa (2/5, 40%), East Africa (3/3, 100%), Southern Africa (6/6, 100%), and West Africa (6/7, 86%).

## Discussion

Integration of HIV and MCH services was reported in all IeDEA regions, with East and Southern Africa reporting the highest proportions of fully integrated services. In addition, we identified country-level policies recommending HIV and MCH integration for all countries in the East and Southern Africa IeDEA regions. East and Southern Africa have the highest burdens of HIV in the world, and account for half of all people with HIV, including two-thirds of all children with HIV (21). It is possible that the high levels of integration in these regions, compared to other regions in our study, are the result of policies and research responsive to their higher burdens of HIV among women and their exposed children, and the need to enhance access to HIV care and treatment services by integrating HIV and MCH services for these populations (1, 15). In Kenya, for example, significant investments in integration research and stakeholder engagement likely played a critical role in promoting integration in Kenya and more broadly in the region (22–25).

Not all sites in East and Southern Africa offered integrated services, however, and there was heterogeneity in the prevalence and characteristics of integration both within and between IeDEA regions in our study. Similar heterogeneity in the uptake of integration was identified in a systematic review conducted in 2010 (4). We also could not identify policies recommending HIV and MCH integration in more than half of countries in CCASAnet and Central Africa. Although, it is possible that those countries lacking an explicit integration policy in Ministry of Health guidance were reliant on WHO or regional guidelines (e.g., Pan American Health Organization) for such policy (1, 26). More research is needed to determine the factors driving integration in LMICs, and the impact of integration on HIV vertical transmission and other maternal and child health outcomes, to understand this heterogeneity and how to measure and address it through WHO and national policy implementation (27).

Several factors could explain the lower proportions of integrated HIV and MCH services outside of East and Southern Africa. In the Asia-Pacific, the lower levels of HIV and MCH integration may relate to this region's higher overall economic status compared to other regions. The IeDEA Asia-Pacific region includes countries from upper-middle (China, Malaysia, Taiwan, Thailand) and high (Austrailia, Japan, South Korea) income strata according to World Bank classifications (28). These countries may have sufficient resources and infrastructure to meet the individual HIV care and treatment needs of WWH and their infants within existing care systems. The lower levels of HIV and MCH integration in the Asia-Pacific could also relate to the nature of this region's epidemic which is largely driven by key populations such as men who have sex with men, sex workers, and people who inject drugs, as opposed to the epidemic in sub-Saharan Africa which disproportionately affects heterosexual women of childbearing potential (21). Therefore, it may be the case that policy implementation and resources in the Asia-Pacific region are primarily focused on these key populations. Nevertheless, some countries, such as the Philippines, are experiencing a resurgence in HIV incidence among women (21). Some countries are also experiencing challenges transitioning from a reliance on international aid to domestic financing of HIV services, particularly in the context of the COVID-19 pandemic. These challenges underscore the need to better understand the advantages of integration in different settings and the impact of country-level policies on integration implementation.

It is also possible that integration is linked in part to a site's geographic setting. In our study, integration was most prevalent at sites serving rural and mixed urban/rural populations compared to urban populations for pregnant and postpartum WWH, and ICEH. Rural clinics may have been better positioned and incentivized to implement integrated HIV and MCH services given more available clinic space or the need to optimize staffing and resource allocation. Patients desiring access to more specialized services and advanced care at urban clinics may also act as a counter force to integration in these settings, compared to rural clinics with less access to these services. Furthermore, the potential association between integration and rural settings was mostly driven by East Africa, which accounted for 37 of 43 (86%) rural sites included in the analysis, whereas sites in other IeDEA regions had relatively higher proportions of urban and mixed urban/rural population settings. The international and national integration policies we reviewed do not distinguish between rural and urban settings. These findings highlight the significant need for further research to understand what is enabling MCH integration in rural areas which may be transportable to other settings.

A strength of our study is its use of the well-established IeDEA-wide site assessment platform to survey global HIV care and treatment sites. This platform enabled us to gather unique global data that have not been previously reported. A potential limitation of our study is that the IeDEA sites included in the analysis may not be generalizable to the broader array of HIV care and treatment sites in the countries represented. For example, sites were initially selected to participate in IeDEA based on their data collection infrastructures, which may imply that they have higher levels of resources, funding and available services compared to non-IeDEA sites. This potential limitation is the reason we elected not to segment the data at the country level. Our data may also be subject to social desirability and reporting biases by clinical staff who completed the surveys, as well as biases arising from the ways the questions were interpreted, for which there was limited ability for investigators to verify responses. Recall bias is also a potential limitation, as respondents were asked to consider practices prior to, rather than during, the COVID-19 pandemic.

Our non-systematic search may have failed to identify relevant ministry of health integration policies for some countries. Country-level guidance on MCH integration can be disseminated through local policy circulars, notices and press releases published years ago that are no longer searchable electronically, so we did not consider it feasible to systematically assess all potentially related policy documents for all countries. Rather, our search was enhanced by direct communications with IeDEA regional and country-level representatives who were able to provide expertise on integration policy for each country. Donor policies and priorities, such as the U.S. President's Emergency Plan for AIDS Relief (PEPFAR), may also have important influences on integration which were not accounted for in our search (17). Still, the findings from our search align with a recent systematic review assessing global HIV and MCH integration research, as well as WHO guidance recommending integration of HIV and other health services particularly in areas with high HIV prevalence (1, 15). Finally, the structured site survey did not allow us to fully tease apart the nuances of service delivery at each site, for example, in Southern Africa, where high proportions of sites reported providing HIV care to pregnant WWH in both the MCH and HIV clinic. In future research involving these sites, we will seek to better understand the factors influencing integration as well as the various nuances of service delivery, including measures of service quality and associations with clinical outcomes for pregnant and postpartum WWH and ICEH.

## Conclusion

The integration of HIV and MCH services was most prevalent in East and Southern Africa, regions with the highest burdens of HIV disease and prevalence among women globally. However, there was heterogeneity in the prevalence of integration both within and between regions. More research is needed to understand the factors driving integration of HIV and MCH services, and the impacts of integration on vertical transmission and other maternal and infant outcomes, to inform how best to address the gaps in integration implementation. The findings of our study will be useful to policymakers interested in the impact of integration policy and its global implementation, as well as program stakeholders and researchers concerned with health system structures and improving service delivery to pregnant and postpartum WWH and their infants.

## Data Availability

Complete data for this study cannot be posted in a supplemental file or a public repository because of legal and ethical restrictions. The Principles of Collaboration under which the IeDEA multiregional collaboration was founded and the regulatory requirements of the different countries' IRBs require the submission of a project concept proposal and approval by the IeDEA Executive Committee. To request data, please review IeDEA guidance and complete the concept proposal template, available at: https://www.iedea.org/resources/multiregional-research-sops-templates/. Signing of a data sharing agreement may also be required. Requests to access the datasets should be directed to humphrjm@iu.edu.
