# Supplementary material for: Integration of HIV care into maternal and child health services in the global IeDEA consortium
Source: Front Glob Womens Health. 2023 Apr 17;4:1066297. doi: 10.3389/fgwh.2023.1066297 (PMC10150067; doi:10.3389/fgwh.2023.1066297)
Supplement: Supplementary file 1 [file Table1.docx]

**SUPPLEMENTARY MATERIAL**

**Table S1**. Integration of HIV and maternal and child health services, by urban and rural population setting, September 2020 to March 2021.

| **Integration status** | **Urban**  **N (%)** | **Rural**  **N (%)** | **Mixed urban/rural**  **N (%)** |
| --- | --- | --- | --- |
| **Pregnant WWH** | **N=46** | **N=43** | **N=89** |
| Full | 20 (43) | 32 (74) | 45 (50) |
| Partial | 11 (24) | 3 (7) | 23 (26) |
| None | 15 (33) | 8 (19) | 21(24) |
| **Postpartum WWH** | **N=44** | **N=41** | **N=84** |
| Full | 13 (30) | 32 (78) | 41 (49) |
| Partial | 5 (11) | 2 (5) | 9 (11) |
| None | 26 (59) | 7 (17) | 34 (40) |
| **ICEH** | **N=44** | **N=41** | **N=74** |
| Full | 17 (39) | 33 (80) | 39 (53) |
| Partial | 5 (11) | 2 (5) | 9 (12) |
| None | 22 (50) | 6 (15) | 26 (35) |

**Table S2.** Evidence of country policies promoting integration of HIV and maternal and child health services.

| **IeDEA Region** | **Country** | **Integration policy** | **Source** |
| --- | --- | --- | --- |
| Asia-Pacific | Australia | Yes | Australasian Society for HIV, Viral Hepatitis and Sexual Health Medicine (ASHM) Sub-Committee for Guidance on HIV Management in Australia. Sydney, 2022. Available from: https://arv.ashm.org.au. Accessed August 16, 2022. |
|  | Cambodia | Yes | Cambodia Ministry of Health. National road map for the elimination of mother-to-child transmission of HIV and congenital syphilis (eMTCT) in Cambodia. Phnom Pehn, 2018. Available from: https://www.aidsdatahub.org/sites/default/files/resource/cambodia-emtct-road-map-2018.pdf. Accessed August 17, 2022. |
|  | China | Yes | China Ministry of Health. Report on Women and Children’s Health Development in China. Beijing, 2011. Available from: http://www.gov.cn/gzdt/att/att/site1/20110921/001e3741a4740fe3bdbf02.pdf. Accessed August 16, 2022.  Huang Z, Jin M, Zhou H, et al. The uptake of prevention of mother-to-child HIV transmission programs in China: A systematic review and meta-analysis. PLoS One. 2015 Aug 26;10(8):e0135068. |
|  | India | Yes | Government of India Ministry of Health and Family Welfare. National Technical Guidelines on Anti Retroviral Treatment. New Delhi, 2018. Available from: https://lms.naco.gov.in/frontend/content/NACO%20-%20National%20Technical%20Guidelines%20on%20ART_October%202018%20(1).pdf. Accessed August 16, 2022. |
|  | Indonesia | Yes | Indonesia Ministry of Health. Program guidelines for the prevention of mother-to-child transmission of HIV, syphilis and hepatitis B, 2019. Available from: https://siha.kemkes.go.id/portal/files_upload/Isi_Buku_PPIA.pdf . Accessed September 8, 2022.  Lumbantoruan C, Kelaher M, Kermonde M, et al. Pregnant women’s retention and associated health facility characteristics in the prevention of mother-to-child HIV transmission in Indonesia: cross-sectional study. BMJ Open. 2020 Sep 24;10(9):e034418. |
|  | Japan | Yes | Japan Ministry of Health, Labour and Welfare. [website]. Guidelines for maternal HIV testing, PMTCT, and care for children living with HIV in Japan, 2022. Available from: http://hivboshi.org/. Accessed September 8, 2022.  Japan Ministry of Health, Labour and Welfare. Guidelines for maternal HIV testing, PMTCT, and care for children living with HIV in Japan, 2022. Available from: http://hivboshi.org. Accessed September 8, 2022.  Japan Ministry of Health, Labour and Welfare. Clinical guidelines for HIV-infected pregnancy and prevention of mother-to-child transmission of HIV, 2021. Available from: http://hivboshi.org. Accessed September 8, 2022. |
|  | Malaysia | Yes | Malaysia Ministry of Health. Elimination of mother-to-child transmission of HIV and syphilis in Malaysia. Putrajaya, 2019. Available from: https://www.moh.gov.my/moh/resources/Penerbitan/Laporan/Umum/CaseStudy-EMTC-lowres5.pdf. Accessed August 17, 2022. |
|  | South Korea | Yes | The Korean Society for AIDS. 2021 Clinical Guidelines for the Diagnosis and Treatment of HIV/AIDS in HIV-infected Koreans. Infect Chemother. 2021 Sep;53(3):592-616. Available from https://doi.org/10.3947/ic.2021.0305.  Korea Disease Control and Prevention Agency. Guidelines for use of antiretrovirals to prevent mother to child transmission of HIV. Osong, 2014. Available from: https://www.kdca.go.kr/board/board.es?mid=a20507020000&bid=0019&act=view&list_no=138241. Accessed August 17, 2022. |
|  | Philippines | Yes | Philippines Department of Health. AO 2022-24 Guidelines on Differentiated Treatment for People Living with Human Immunodeficiency Virus (PLHIV) and Prophylaxis for HIV-Exposed Infants, 2022. Available from: https://www.ship.ph/ao-2022-24-guidelines-on-differentiated-treatment-for-people-living-with-human-immunodeficiency-virus-plhiv-and-prophylaxis-for-hiv-exposed-infants/. Accessed September 8, 2022. |
|  | Taiwan | Yes | Taiwan Centers for Disease Control. Prevention of vertical infection of mother and child [website; translated]. Available from: https://www.cdc.gov.tw/Category/MPage/gNNbeV3RF6cJNHhDBOOxrQ. Accessed September 8, 2022.  Huang Z, Jin M, Zhou H, et al. The uptake of prevention of mother-to-child HIV transmission programs in China: A systematic review and meta-analysis. PLoS One. 2015 Aug 26;10(8):e0135068. |
|  | Thailand | Yes | Thailand Ministry of Public Health. Thailand National Guidelines on HIV/AIDS Treatment and Prevention 2021/2022. Available from: http://www.thaiaidssociety.org/images/PDF/hiv-aids-guideline-2564_2565.pdf. Accessed September 6th, 2022. |
|  | Vietnam | Yes | Vietnam Ministry of Health. Decision on issuance of guidelines for HIV/AIDS treatment and care. Number 5968/QD-BYT. Hanoi, December 31,2021.  Vietnam Ministry of Health. National guidelines for care, treatment and support of HIV infected pregnant women, children and HIV-infected women. Decision 872/QD-BYT. March 19th, 2013.  Le CT, Vu TT, Luu MC, et al. Preventing mother-to-child transmission of HIV in Vietnam: An assessment of progress and future directions. J Trop Pediatr. 2008 Aug;54(4):225-32. |
| CCASAnet | Argentina | Unconfirmed | Argentina Ministry of Health. [website]. Available from: https://www.argentina.gob.ar/salud/vih-sida. Accessed August 17, 2022. |
|  | Brazil | Yes | Brasil. Ministério da Saúde. Secretaria de Vigilância em Saúde. Departamento de Doenças de Condições Crônicas e Infecções Sexualmente Transmissíveis. Protocolo Clínico e Diretrizes Terapêuticas para Prevenção da Transmissão Vertical do HIV, Sífilis e Hepatites Virais / Ministério da Saúde, Secretaria de Vigilância em Saúde, Departamento de Doenças de Condições Crônicas e Infecções Sexualmente Transmissíveis. – Brasília : Ministério da Saúde, 2019. Available from: http://www.aids.gov.br/system/tdf/pub/2016/57801/miolo_pcdt_tv_08_2019.pdf?file=1&type=node&id=57801&force=1. Accessed September 9, 2022. |
|  | Chile | Unconfirmed | Chile Ministry of Health. [website]. Available from: https://diprece.minsal.cl/le-informamos/auge/acceso-guias-clinicas/guias-clinicas-auge/. Accessed August 17, 2022. |
|  | Haiti | Yes | Lepira L, Kemp C, Domercant J, et al. The role of service readiness and health care facility factors in attrition from Option B+ in Haiti: a joint examination of electronic medical records and service provision assessment survey data. Int Health. 2018 Jan; 10(1): 54–62. |
|  | Honduras | Unconfirmed | Honduras Ministry of Health. [website]. Tegucigalpa, 2022. Available from: http://www.salud.gob.hn/site/. Accessed August 18, 2022. |
|  | Mexico | Unconfirmed | Mexico Ministry of Health. [website]. Acapulco, 2022. Available from: https://www.gob.mx/salud. Accessed August 18, 2022. |
|  | Peru | Unconfirmed | Peru Ministry of Health. [website]. Lima, 2022. Available from: https://www.gob.pe/minsa. Accessed August 18, 2022. |
| Central Africa | Burundi | Unconfirmed | Burundi Ministère de la Santè Publique et de la Lutte Contre le SIDA. Directives nationales d’utilisation des antiretroviraux pour la prevention et le traitement du VIH. Bujumbura, 2016. Available from: http://minisante.bi/wp-content/uploads/pnls/Nouvelles%20Directives%202016.pdf. Accessed August 18, 2022. |
|  | Cameroon | Yes | Cameroon Ministry of Public Health. National guideline on the prevention and management of HIV in Cameroon. Yaounde, 2015. Available from: https://www.childrenandaids.org/sites/default/files/2018-05/Cameroon_Nat%20Guidelines%20HIV_2015.pdf. Accessed August 18, 2022. |
|  | Democratic Republic of Congo | Unconfirmed | République Démocratique du Congo Ministère de la santé publique. Guide de prise en charge intégrée du VIH en République Démocratique du Congo. Kinshasa, 2016. Available from: https://www.prepwatch.org/wp-content/uploads/2022/03/DRC-National-PrEP-Guideliens-2019.pdf. Accessed August 18, 2022.  République Démocratique du Congo Ministère de la santé publique. Plan opérationnel d’offre des services différenciés en République Démocratique du Congo. Kinshasa, 2018. Available from: https://differentiatedservicedelivery.org/Portals/0/adam/Content/BJwmFXyVAUmyjxMNTDqIgg/File/RDC%20Plan%20opérationnel%20soins%20différenciés%20V%2020%2008%202018.pdf. Accessed August 18, 2022. |
|  | Republic of Congo | Unconfirmed | None |
|  | Rwanda | Yes | Rwanda Ministry of Health. National guidelines for prevention and management of HIV and STIs. Kigali, 2016. Available from: https://rbc.gov.rw/fileadmin/user_upload/guide/SIGNED%20ENGLISH%20%202016%20VERSION.pdf. Accessed August 17, 2022. |
| East Africa | Kenya | Yes | Kenya Ministry of Health. Guidelines on the use of antiretroviral drugs for treating and preventing HIV in Kenya. Nairobi, 2018. Available from: https://www.nascop.or.ke/new-guidelines/. Accessed August 8, 2022. |
|  | Tanzania | Yes | Tanzania Ministry of Health, Community Development, Gender, Elderly, and Children. National Guidelines for the Management of HIV and AIDS. Dodoma, 2019. Available from: https://nacp.go.tz/download/national-guidelines-for-the-management-of-hiv-and-aids/. Accessed August 8, 2022. |
|  | Uganda | Yes | Uganda Ministry of Health. Consolidated guidelines for prevention and treatment of HIV in Uganda.  Kampala, 2016. Available from: http://library.health.go.ug/publications/hivaids/consolidated-guidelines-prevention-and-treatment-hiv-uganda. Accessed August 8, 2022. |
| Southern Africa | Lesotho | Yes | Lesotho Ministry of Health. National Guidelines on the use of antiretroviral therapy for HIV prevention and treatment. Maseru, 2016. Available from: https://www.childrenandaids.org/sites/default/files/2017-04/Lesotho_ART-Guidelines_2016.pdf. Accessed August 8, 2022. |
|  | Malawi | Yes | Malawi Ministry of Health. Clinical Management of HIV in Children and Adults. Lilongwe, 2014. Available from: https://www.differentiatedservicedelivery.org/Portals/0/adam/Content/yb4xSSLvE0SW98_z7wTm_w/File/Malawi%20Clinical%20HIV%20Guidelines%202018%20(1).pdf. Accessed August 8, 2022. |
|  | Mozambique | Yes | Mozambique Ministry of Health. Guião Orientador sobre Modelos Diferenciados de Serviços em Moçambique [Guidance on Differentiated Service Models in Mozambique]. Maputo, 2018. Available from: https://differentiatedservicedelivery.org/Guidance/National-policy. Accessed August 8, 2022. |
|  | South Africa | Yes | South African National Department of Health. Guideline for the prevention of mother to child transmission of communicable infections. Pretoria, 2019. Available from: https://www.knowledgehub.org.za/system/files/elibdownloads/2019-10/PMTCT%20Guideline%2028%20October%20signed.pdf. Accessed August 8, 2022. |
|  | Zambia | Yes | Zambia Ministry of Health. Zambia consolidated guidelines for treatment and prevention of HIV infection. Lusaka, 2016. Available from: https://www.childrenandaids.org/sites/default/files/2017-04/Zambia_Consolidated-HIV-Guidelines_2016.pdf. Accessed August 8, 2022. |
|  | Zimbabwe | Yes | Zimbabwe Ministry of Health and Child Care. Operational and service delivery manual for the prevention, care and treatment of HIV in Zimbabwe. Harare, 2017. Available from: http://www.ophid.org/treat-all-toolkit/MOHCC%20Guidelines/MOHCC%20Zimbabwe%20OSDM%202017.pdf. Accessed August 8, 2022. |
| West Africa | Benin | Yes | Bènin Ministère de la Santè. [website]. Porto Novo, 2022. Available from: https://sante.gouv.bj/PNLS-Programme-National-de-Lutte-contre-le-Sida. Accessed August 8, 2022. |
|  | Burkina Faso | Yes | World Health Organization. Rapid Assessment of Sexual and Reproductive Health and HIV Linkages. Geneva, 2013. Available from: http://srhhivlinkages.org/wp-content/uploads/2013/04/rasburkinafaso_2011_en.pdf. Accessed August 18, 2022. |
|  | Cote d’Ivoire | Yes | Kouamé HPA, Dogbo ND, Touré S, et al. PMTCT activities implementation: case of Côte d'Ivoire, from ACONDA's experience. Retrovirology 2009;6(Suppl 1):O17. Available from: https://retrovirology.biomedcentral.com/track/pdf/10.1186/1742-4690-6-S1-O17.pdf. Accessed August 18, 2022. |
|  | Ghana | Yes | Ghana National AIDS/STI Control Programme. 2011 Annual Report. Accra, 2012.  Dako-Gyeke P, Dornoo B, Addo SA, et al. Towards elimination of mother-to-child transmission of HIV in Ghana: an analysis of national programme data. Int J Equity Health. 2016 Jan 13;15:5. |
|  | Mali | Unconfirmed | Mali Ministère de la Santè et du Dèveloppement Social. [website]. Bamako, 2022. Available from: http://www.sante.gov.ml/index.php/2014-11-10-17-29-36/documents-politiques22. Accessed August 8, 2022. |
|  | Senegal | Yes | Guide pour la prise en charge de l’infection par le vih au Senegal. Available from: https://differentiatedservicedelivery.org/Portals/0/adam/Content/LVmdpZw8IUWOOAzlez4_2g/File/Senegal_Guide%20PEC%20VIH_4-11-18.docx.pdf. Dakar, 2018. Accessed August 18, 2022. |
|  | Togo | Yes | Ministère de la Santè et de L’Hygiène Publique. Guide de prise en charge globale des personnes vivant avec le VIH Available from: https://differentiatedservicedelivery.org/Portals/0/adam/Content/KitF_OhVeESPHQXlQf-Hrw/File/Togo_HIV%20Clinical%20Guidelines_May%202019.pdf. Lomé, 2019. Accessed August 18, 2022. |
